# Paravertebral block vs. epidural block for percutaneous nephrolithotomy: A prospective, randomized study

**DOI:** 10.3389/fsurg.2023.1112642

**Published:** 2023-03-23

**Authors:** Pengcheng Zhu, Qianqian Meng, YuanYuan Miao, Le Zhou, Chun Wang, Haitao Yang

**Affiliations:** Department of Anesthesiology, The Second Hospital of Dalian Medical University, Dalian, China

**Keywords:** percutaneous nephrostolithotomy, paravertebral nerve block, epidural anesthesia, nephrolithiasis, ERAS

## Abstract

**Background:**

Percutaneous nephrolithotripsy (PCNL) is the main method for urinary calculi. An anesthesia method with little effect on the blood circulation and which does not affect the postoperative activity of a patient is lacking.

**Objective:**

To compare the effects of paravertebral nerve block (PNB) and epidural block (EPB) on quadriceps femoris muscle (QFM) strength in patients after PCNL.

**Methods:**

163 patients were separated into two groups: EPB (81) and PNB (82). Primary outcome parameters were QFM strength and range of motion (RoM) of the knee 1 h, 2 h, 3 h, and 24 h after anesthesia induction (AI). Secondary outcome parameters were: time from AI beginning to first ambulation; time of sensory-plane recovery; amount of additional analgesics given during and after surgery; prevalence of nausea and vomiting; duration of hospital stay (DoHS); mean arterial pressure (MAP), heart rate (HR), and oxygen saturation (SpO_2_) before, 0.5 h, and 1 h after AI; visual analog scale (VAS) score 0.5 h, 1 h, 2 h, 3 h and 24 h after AI.

**Results:**

There was no significant difference in QFM strength or knee RoM before or 24 h after AI between the two groups (*P* > 0.05). The time from AI to first ambulation was shorter (*P* < 0.05) and the sensory plane took longer to recover (*P* < 0.05) in the PNB group than in the EPB group. The amount of additional analgesics during surgery was more in the PNB group than in the EPB group (*P* < 0.05), but there was no significant difference after surgery (*P* > 0.05). VAS scores were higher in the PNB group than in the EPB group 0.5 after AI (*P* < 0.05). MAP 1 h after AI was higher in the PNB group than in the EPB group (*P* < 0.05). There was no significant difference in the prevalence of postoperative nausea and vomiting, DoHS, HR, or SpO_2_ at 0.5 h and 1 h after AI between the two groups (*P* > 0.05).

**Conclusions:**

For patients undergoing PCNL, PNB can meet the need for surgical analgesia while having little effect on QFM strength.

**Trial registration:**

http://www.chictr.org.cn/, identifier ChiCTR2200060606.

## Introduction

Urinary calculi are common urological disorders. In China, the prevalence of urinary calculi is about 1%–5% and the annual incidence is about 150–200/100,000 people, about 25% of whom require hospitalization (1).

In 1976, Fernstrom and Johannson were the first to describe the removal of renal calculi by nephrostomy. With advancements in medical technology and development of devices, percutaneous nephrolithotomy (PCNL) is the preferred method for treatment of larger renal calculi (>20 mm), staghorn calculi, and multiple calculi (2). The main steps are transurethral placement of a double-J tube in the lithotomy position, changing the position of the patient to prone to establish a lithotripsy channel using ultrasound, lithotripsy and sucking the residue out of the body, and nephrostomy (3).

The anesthesia methods employed for PCNL are general anesthesia and combined spinal–epidural anesthesia. The latter has a long induction time (4), and the motor function of both lower limbs disappears after local-anesthetic solution is injected into the subarachnoid space. The patient cannot move onto the ground until the local anesthetic has been metabolized completely. In addition, hypotension, headache, urinary retention, and other complications may occur. General anesthesia has a rapid onset of action, but can have a considerable hemodynamic impact (5). Hypotension, bradycardia, and other complications are likely to occur intraoperatively. Delirium, delayed recovery, chills, nausea, vomiting and other complications can occur postoperatively.

In recent years, the concept of enhanced recovery after surgery (ERAS) has been accepted by many surgeons in China. Ensuring the correct diagnosis and optimal treatment effect while minimizing the duration of hospital stay (DoHS) of patients has become an important concern for physicians.

“Epidural block” (EPB) refers to injection of a local anesthetic into the epidural space, thereby blocking the spinal nerve roots and paralyzing the areas innervated by them temporarily. EPB is segmental and can be sufficient for PCNL. However, the drug can spread to bilateral spinal nerve roots after epidural injection, and its blocking effect is bilateral. Simultaneously, EPB may also affect the strength of the unaffected quadriceps femoris muscle (QFM) due to the downward spread of the drug, thereby resulting in delayed postoperative ambulation and prolonged hospitalization (6).

“Paravertebral nerve block” (PNB) refers to injection of a local anesthetic into the paravertebral space and blockade of somatic nerves in consecutive multiple dermatomal areas on one side of the body (7). PNB can be administered from T11 to T12, and blockade can spread from T9 to L2. PNB can blunt the conduction of visceral pain and skin-puncture pain in the kidney (8).

We compared the QFM strength and analgesic effect between EPB and PNB in patients undergoing PCNL. In this way, we aimed to provide a reference for clinical practice.

## Materials and methods

### Inclusion criteria

The inclusion criteria were: (i) American Society of Anesthesiologists (ASA) class I–III; (ii) no contraindications to EPB or PNB; (iii) age of 21–70 years.

### Exclusion criteria

The exclusion criteria were: (i) refusal to undergo surgery or be anesthetized; (ii) hypersusceptible to local anesthetics; (iii) coagulopathy; (iv) tumor or contamination at the puncture site; (v) spinal deformity; (vi) disease affecting the central nervous system; (vii) mental illness.

### Study cohort

163 patients with renal calculi requiring PCNL admitted to the Second Hospital of Dalian Medical University from June 2022 to October 2022 were selected for this study.

### Patient grouping

All patients were separated into two groups based on a random number form: EPB and PNB.

### Procedure

At the preoperative visit, the anesthesiologist explained the visual analog scale (VAS), the surgical procedure, as well as EPB and PNB procedures to the patient. VAS is a simple and common scale to assess the degree of pain and widely used in perioperative pain assessment. The total score is 10, zero for no pain and 10 for severe pain. On a scale of 0 to 10. The higher the number, the worse the pain. In order to alleviate the anxiety of patients, 0.05 mg/kg midazolam was injected through vein for anti-anxiety in the ward half an hour before surgery. Noninvasive measurement of blood pressure, mean arterial pressure (MAP), electrocardiography, and oxygen saturation (SpO_2_) were monitored after admission to the operating theatre. Baseline parameters were recorded, and venous access was established using a 16-G or 18-G needle. Before the induction of anesthesia, an anesthesiologist assessed the QFM strength by a freehand method, and range of motion of the knee was also measured.

Patients in the EPB group were selected for puncture at T11 and T12 spaces. The epidural catheter was placed 3 cm–4 cm downward and fixed after epidural puncture. After catheterization, 2% lidocaine (5 ml) was administered as an experimental dose. The plane of anesthesia was tested 5 min after confirmation that the patient had not suffered an adverse reaction (e.g., total spinal anesthesia or poisoning by local anesthetic). The anesthetist injected 0.5% ropivacaine (7–10 ml) through the catheter. The procedure started 15 min later, when the anesthesia plane reached T6–L2. If the anesthesia plane had not been reached, the method was changed to general anesthesia and experimental data removed.

Patients from the PNB group were placed in the lateral position with their heads lowered and bent over. A linear array probe (5–10 MHz) was used at the T11–T12 gap. When clear images of the transverse process were seen, 1% lidocaine was used to locally infiltrate the puncture site and aid insertion of needles in the plane. Needles were inserted into the paravertebral space under real-time ultrasound guidance. The anesthetist injected 0.5% ropivacaine (15–20 ml) after no blood was withdrawn from the syringe. PNB block was considered successful if, 15 min later, the pleura moved downwards during injection and the acupuncture block level covered T10 to L2. The urethra was anesthetized topically with 1% tetracaine (5 ml) injected from the urethra before the start of surgery. Each patient in both groups group complained of intraoperative pain, so oxycodone (2–5 mg, i.v.) was given and the total amount of oxycodone used by each patient recorded. Postoperatively, patients (with monitoring) were sent to the Post-Anesthesia Care Unit. Postoperative pain was assessed with VAS, and pain intensity was recorded 0.5 h, 1 h, 2 h, 3 h and 24 h after anesthesia induction (AI). If VAS >3, then oxycodone (2–5 mg) was administered.

### Outcome measures

The primary outcome parameters were QFM strength and RoM of the knee 1 h, 2 h, 3 h, and 24 h after AI.

The secondary outcome parameters were: time from the beginning of anesthesia to first ambulation; time of sensory-plane recovery; amount of additional analgesics given during and after surgery; prevalence of nausea and vomiting; DoHS; MAP, HR, and SpO_2_ before, 0.5 h and 1 h after AI; VAS score 0.5 h, 1 h, and 2 h, 3 h, and 24 h after AI.

### Statistical analyses

PASS 15.0 (NCSS, NY, USA) was used to estimate sample size. Twenty patients were included in the pilot study and divided randomly into two groups. The mean grade of QFM strength was 2.70 (SD = 0.98) in the EPB group and 3.50 (SD = 0.70) in the PNB group. We used 90% statistical power and the test level was *α* = 0.05 (bilateral). Considering a 20% loss to follow-up or withdrawal from our study, we calculated that 160 patients were needed in this study.

SPSS 25.0 (IBM, Armonk, NY, United States) was employed to process and analyze data. Enumeration data are expressed as percentages. Measurement data are expressed as the mean ± SD. Measurement data of skewed distribution are presented as median (M) and interquartile range (IQR). Differences between the two groups were compared by the Student's *t*-test. Categorical variables were analyzed by the chi-square test. Wilcoxon rank sum test was used to compare grade data between groups. *P* < 0.05 was considered significant.

## Results

The EPB group comprised 81 patients, one of whom was excluded from the analysis due to circulatory instability after anesthesia, and the data of two patients in the PNB group were deleted because the plane of anesthesia did not meet surgical requirements. Finally, the data of 80 patients in the EPB group and 80 patients in the PNB group were analyzed. There were no obvious differences in demographic parameters, operative duration, blood loss, or ASA score between the two groups (Table 1).

**Table 1:** Characteristics of patients in the two groups.

	EPB group (*n* = 80)	PNB group (*n* = 80)	*P*
Age (years)	57.58 ± 9.61	56.1 ± 9.5	0.293
% Males	55.0%	47.5%	>0.05
Operative time (min)	52.99 ± 12.87	54.40 ± 13.42	0.498
Blood loss (ml)	33.43 ± 9.73	34.69 ± 9.26	0.406
ASA score (II%)	85.0%	82.5%	>0.05

ASA, American Society of Anesthesiologists.

A significant difference in QFM strength or RoM of the knee was not observed before AI in the two groups (*P* > 0.05) (Table 2). The variation trend of QFM strength and RoM of the knee are revealed in Tables 2, 3. The QFM strength and RoM of the knee decreased 1 h, 2 h, and 3 h after AI in both groups. However, patients in the PNB group had significantly higher QFM and RoM of the knee (Table 3) than the EPB group (*P* < 0.05) 1 h, 2 h, and 3 h after AI. At 24 h after surgery, a meaningful difference in QFM strength and RoM of the knee between the EPB group and PNB group was not observed (*P* > 0.05).

**Table 2:** Strength of quadriceps femoris muscle in the two groups.

	Preoperative	1 h after AI	2 h after AI	3 h after AI	24 h after AI
EPB group	5 (4–5)	2 (1–3)	3 (2–4)	3 (2–4)	5 (4–5)
PNB group	5 (4–5)	4 (3–5)	5 (3–5)	5 (4–5)	5 (4–5)
*P*	0.441	<0.05	<0.05	<0.05	0.135

AI, anesthesia induction.

**Table 3:** Range of motion of the knee (°) in the two groups.

	Preoperative	1 h after AI	2 h after AI	3 h after AI	24 h after AI
EPB group	116.38 ± 4.89	61.44 ± 10.31	76.40 ± 12.08	81.53 ± 10.72	117.34 ± 6.64
PNB group	117.01 ± 5.36	98.54 ± 10.35	109.13 ± 9.19	114.74 ± 5.31	118.80 ± 4.18
*P*	0.441	<0.05	<0.05	<0.05	0.062

AI, anesthesia induction.

The time from the beginning of AI to the first ambulation in the PNB group was shorter (*P* < 0.05). However, the time of sensory-plane recovery in the PNB group was longer than that in the EPB group (*P* < 0.05). Patients in the PNB group consumed more additional analgesics than the EPB group (*P* < 0.05) but the amount of additional postoperative analgesics was similar in both groups (*P* > 0.05). There was no notable difference in the prevalence of postoperative nausea, vomiting, or DoHS in the two groups (Table 4).

**Table 4:** Operative and postoperative features in the two groups.

	EPB group	PNB group	*P*
Time until first ambulation (min)	347.38 ± 33.06	113.94 ± 20.01	<0.05
Time for sensory-plane recovery (min)	419.25 ± 44.71	480.25 ± 24.60	<0.05
Intraoperative oxycodone dose (mg)	2.79 ± 1.85	4.60 ± 2.68	<0.05
Postoperative oxycodone dose (mg)	1.84 ± 1.47	1.56 ± 1.48	0.239
Nausea and vomiting (%)	10%	8.6%	>0.05
Duration of hospital stay (days)	4.04 ± 0.86	3.91 ± 0.84	0.356

The VAS score in the PNB group was higher than that in the EPB group 0.5 h after AI (*P* < 0.05), but it was similar 1 h, 2 h, 3 h and 24 h after AI (*P* > 0.05). A significant difference in HR or SpO_2_ at 0.5 h and 1 h after AI was not observed between the two groups (*P* > 0.05). MAP at 1 h after AI was higher in the PNB group than that in the EPB group (*P* < 0.05) (Tables 5–8).

**Table 5:** Mean arterial pressure (mmHg) in the two groups.

	Preoperative	0.5 h after AI	1 h after AI
EPB group	90.71 ± 8.50	74.92 ± 7.00	75.63 ± 6.86
PNB group	92.74 ± 9.02	85.90 ± 9.13	83.18 ± 7.87
*P*	0.141	<0.05	<0.05

AI, anesthesia induction.

**Table 6:** Oxygen saturation (%) in the two groups.

	Preoperative	0.5 h after AI	1 h after AI
EPB group	98.55 ± 1.29	98.83 ± 1.84	98.91 ± 1.16
PNB group	98.50 ± 1.34	98.41 ± 1.37	98.44 ± 1.12
*P*	>0.05	>0.05	>0.05

AI, anesthesia induction.

**Table 7:** Heart rate (bpm) in the two groups.

	Preoperative	0.5 h after AI	1 h after AI
EPB group	79.50 ± 8.26	75.78 ± 5.85	75.77 ± 5.13
PNB group	81.33 ± 9.31	77.89 ± 6.16	77.20 ± 6.06
*P*	0.189	0.064	0.111

AI, anesthesia induction.

**Table 8:** VAS score in the two groups.

	0.5 h after AI	1 h after AI	2 h after AI	3 h after AI	24 h after AI
EPB group	3 (1–5)	2 (0–3)	1 (0–2)	1 (0–1)	1 (0–1)
PNB group	3 (2–6)	2 (1–3)	1 (0–2)	1 (0–2)	1 (0–1)
*P*	<0.05	0.442	0.424	0.497	0.554

AI, anesthesia induction.

## Discussion

PCNL is a safe, minimally invasive, and efficacious therapy for complex stones in the upper urinary tract. Patients with urinary stones can avoid surgical injuries and recover sooner if PCNL is used compared with other procedures.

The sympathetic nerves of the kidney originate from T10 to L1 (9). Sympathetic nerves in the bladder originate from T12 to L2. The sensory nerves of the kidney and bladder are accompanied by a corresponding sympathetic course. The QFM lies in front of the thigh muscles and is innervated mainly by the femoral nerve (L2–L4). Theoretically, adjusting the block plane to T10–L2 can meet the anesthetic needs of PCNL without affecting QFM contraction. However, the epidural space is an upper and lower interconnecting space (10). A small proportion of local anesthetics may flow from the T11–T12 space to the foot, thereby making the plane of anesthesia slightly more than L2 and affecting QFM strength (11). This effect is bilateral, so most of the bilateral QFM strength in the EPB group was grade 2–3 at 1 h and 2 h after AI. Local anesthetics injected into the paravertebral space also flow caudally and affect QFM strength due to the presence of the thoracolumbar fascia (12). However, this effect is unilateral. The nerve distribution here is more scattered than that in the spinal canal. Hence, the strength of the affected QFM was higher, and the QFM strength of the unaffected side was barely impacted in patients in the PNB group compared with those in the EPB group 1 h and 2 h after AI. Patients in the PNB group also had a shorter time to recover QFM strength to grades 4–5 than those in the EPB group. Patients also took a significantly shorter time to ambulate than the EPB group. A meta-analysis by Tan et al. showed that carrying out PNB in PCNL helped to shorten the DoHS (13). At 24 h after AI, the effects of EPB and PNB had subsided completely, and there was no residual effect on the lower-limb movement of patients. The lower-limb muscle strength and RoM of the knee of patients in both groups could return to a normal level before surgery.

The paravertebral space is relatively enclosed, so injected local anesthetics can spread down through the thoracolumbar fascia and affect nerves at L2 or lower. However, the amount of local anesthetic flowing below was small, so the effect was unilateral. The epidural space is connected upwards and downwards, and local anesthetics injected into the epidural space can flow through changes in patient position. At the same time, the effects of local anesthetics were bilateral. In summary, the time to first ambulation in the PNB group could be significantly shorter than that in the EPB group. Simultaneously, due to the relative closure of the paravertebral space, the absorption of local anesthetics was slower, so the effect of local anesthetics was longer, which resulted in a significantly longer recovery time of the sensory plane in the PNB group than that in the EPB group.

All patients in EPB group reached a T10–L2 level of sensory block. Three patients in the EPB group had a reduction in blood pressure which returned to normal after administration of ephedrine (3–6 mg) and accelerated fluid replacement. One patient in the PNB group developed hypotension. All patients with hypotension had no adverse consequences after remedial measures were undertaken.

The PNB group used more oxycodone intraoperatively compared with the EPB group. This phenomenon may have been because a PNB combined with topical anesthesia (1% tetracaine) could not block the stimulation completely during transurethral catheterization. However, the dose was increased by only ∼5 mg, which was within a clinically safe dose range. The oxycodone dose between the two groups after surgery was similar, which indicated that PNB and EPB had similar postoperative analgesic effects. Three patients from the PNB group had higher oxycodone consumption 0.5 h after AI than those in the EPB group, but a significant difference was not seen after surgery. PNB means blocking nerve conduction in paravertebral intervals (in which spinal nerves cross the intervertebral foramen to form lateral spinal nerves) using local anesthetics. PNB has a slow onset of action, and full blockade of spinal nerves to produce anesthesia takes over 15 min. The procedure started 17 min, 18 min, and 15 min after AI in the three cases, respectively. Possibly, the dosage of oxycodone was higher than EPA due to incomplete block. In addition, one patient had an operative time of 1 h and 47 min, and the oxycodone dose in this patient was 13 mg. These data suggest that the patient may have suffered pain due to a prolonged operative time and reduced anesthesia from PNB. An indwelling catheter during PNB may be beneficial for addressing this problem.

Eight patients in the PNB group and six in the PNB were classified as ASA III. Compared with the EPB group, the MAP of cases in the PNB group decreased less after AI than before AI, and there was no significant difference in the HR. No difference was found between ASA-III patients from the two groups with regard to the oxycodone dose, prevalence of nausea and vomiting, VAS score, or DoHS during or after surgery. These data suggest that PNB before PCNL may have higher safety and similar blockade effects than EPB in patients with serious complications. Baldea et al. concluded that PNB for PCNL leads to a stable circulation and does not increase the risk of adverse intraoperative complications (e.g., bleeding, pleural puncture) (14).

Patients in the two groups had a similar mean DoHS of 3–4 days, which is shorter than that for patients who underwent PCNL under general anesthesia in our hospital (∼5 days). This difference may because PNB and EPB can promote early eating and early ambulation of patients, and is associated with fewer postoperative complications.

Our study had four main limitations. First, it was a single-center study. Second, the number of patients in each group was small. Third, the anesthesia methods in the two groups were quite different, so the anesthetist knew the study protocol, which lead to a bias. Fourth, the age range in both groups was wide, and patients were not stratified by age.

## Conclusions

For patients undergoing PCNL, PNB can meet the need for surgical analgesia while having little effect on QFM strength. These features help patients to ambulate early without increasing the risk of postoperative adverse reactions and having less effect upon the blood circulation than EPB.

## Data Availability

The raw data supporting the conclusions of this article will be made available by the authors, without undue reservation.
